# Supplementary material for: The transitional kinetics between open and closed Rep structures can be tuned by salt via two intermediate states
Source: Nucleic Acids Res. 2026 Jan 21;54(2):gkaf1483. doi: 10.1093/nar/gkaf1483 (PMC12820531; doi:10.1093/nar/gkaf1483)
Supplement: gkaf1483_Supplemental_Files [file gkaf1483_supplemental_files.zip › Rep-SI_only_v43_resubmission.pdf]

Supplementary Information

The transitional kinetics between open and closed Rep structures can be tuned by salt via two intermediate states

Jamieson A L Howard[1], Benjamin Ambrose[2,3], Mahmoud A S Abdelhamid[2], Lewis Frame[1], Antoinette Alevropoulos-Borrill[1], Ayesha Ejaz[4], Lara Dresser[1], Maria Dienerowitz[5], Steven D Quinn[1,6], Allison H Squires[4,7], Agnes Noy[1,6], Timothy D Craggs[2], and Mark C Leake[1,6,8 †]

[1] School of Physics, Engineering and Technology, University of York, York, YO10 5DD, UK

[2] Department of Chemistry, University of Sheffield, Sheffield S3 7HF, U.K.

[3] Current address: Single Molecule Imaging Group, MRC-London Institute of Medical Sciences, London, W12 0HS, UK.

[4] Pritzker School of Molecular Engineering, University of Chicago, IL, USA.

[5] SciTec Department, Ernst-Abbe-Hochschule, University of Applied Sciences, Jena, Germany.

[6] York Biomedical Research Institute, University of York, York, YO10 5DD, UK.

[7] Institute for Biophysical Dynamics, University of Chicago, Chicago, IL, USA.

[8] Department of Biology, University of York, York, YO10 5DD, UK.

[†] For correspondence. Email mark.leake@york.ac.uk

Supplementary Information includes:

9 Supplementary Figures

6 Supplementary Tables

7 Supplementary Movie Legends

Supplementary Figures

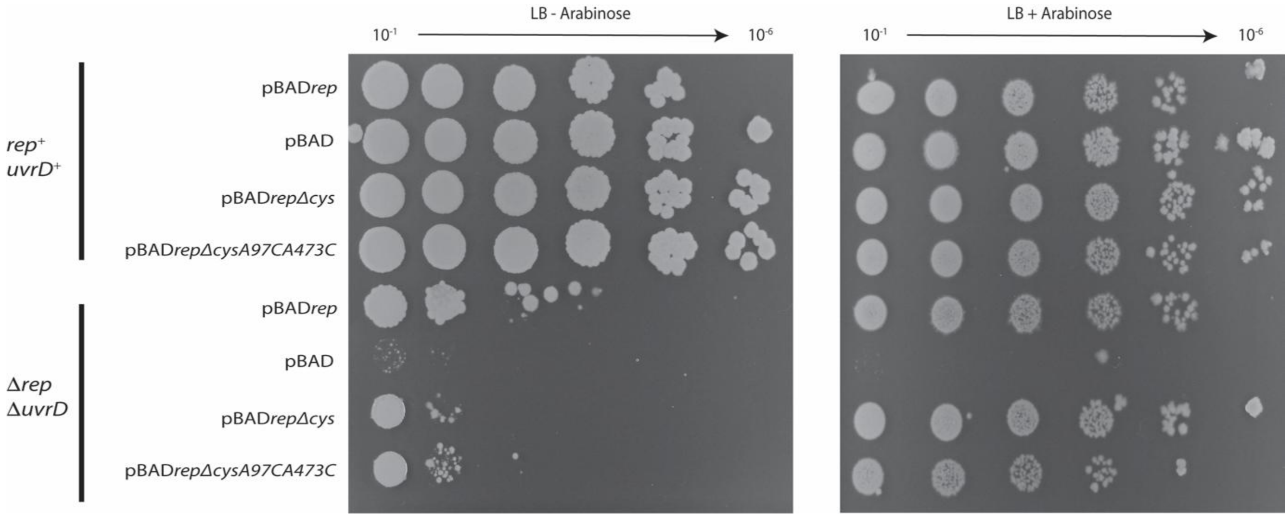

Supplementary Figure 1. Complementation assays reveal that *repΔcysA97CA473C* is functional. Two complementation assays are shown in the absence (left) and presence (right) of arabinose which the synthetic lethality of a *Δrep ΔuvrD* can be rescued by a covering plasmid containing *repΔcysA97CA473C*. When grown on rich media the *Δrep ΔuvrD* is lethal due to conflicts in DNA replication (see lack of growth in *Δrep ΔuvrD* pBAD spots). This phenotype can be rescued by a covering plasmid overexpressing Rep, growth is restored to normal level in *Δrep ΔuvrD* pBAD*rep* in the presence of arabinose. This synthetic lethality is also rescued by covering plasmids overexpressing RepΔcys or RepΔcysA97CA473C, growth is restored to normal levels in in *Δrep ΔuvrD* pBAD*repΔcys and in Δrep ΔuvrD pBADrepΔcysA97CA473C.*

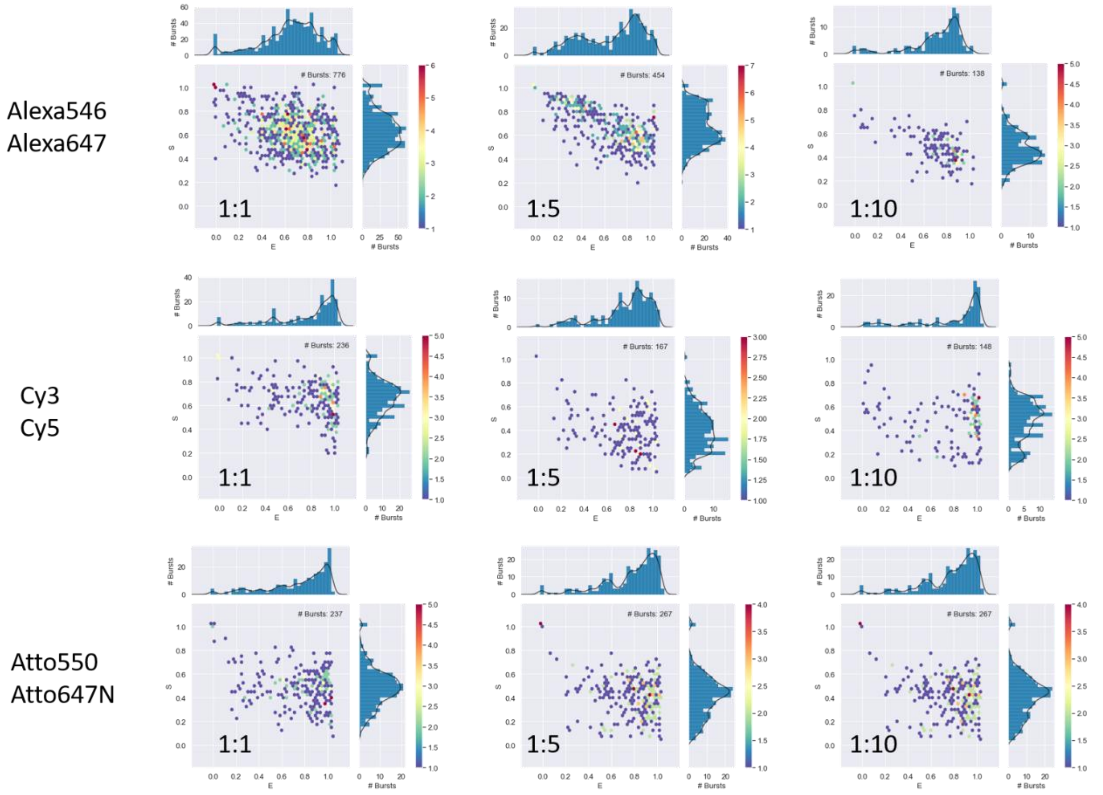

Supplementary Figure 2. Different FRET dye pairs were triaged using confocal smFRET. Using alternating laser excitation, we characterised the E-S relationships of several candidate FRET dye pairs over a range of relative concentrations, indicating that using Alexa Fluor 546 and Alexa Fluor 647 as a dye pair in a 1:1 labelling ration yielded the largest number of bursts using confocal smFRET as well as showing predominantly species containing both a donor and acceptor dye (stoichiometry parameter S= ~0.5, top left plot). Other dyes yielded fewer bursts for a given protein concentration, suggesting either poorer labelling efficiency or the presence of larger aggregates that are not seen in confocal smFRET. It is also clear that other dye pairs and dye ratios led to more donor or acceptor only bursts (S is approximately 1 or 0 respectively), which is sub optimal for smFRET measurements.

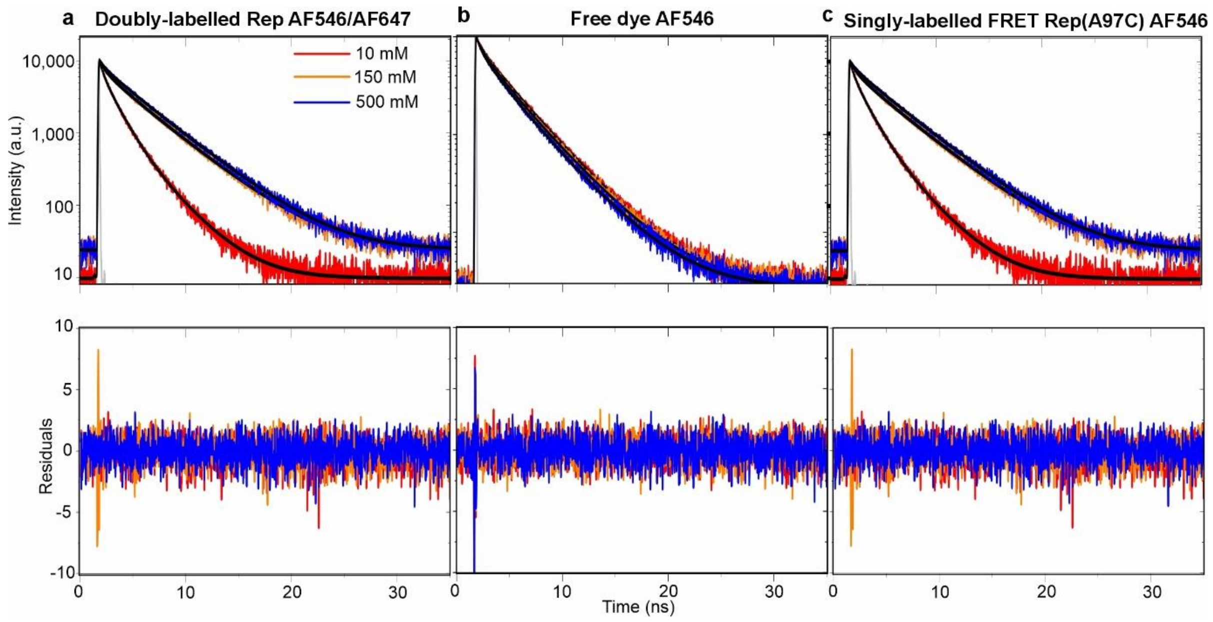

Supplementary Figure 3. Ensemble FRET spectroscopy demonstrates dependence of FRET lifetime on NaCl concentration. Fluorescence intensity decay profiles of **a.** doubly-labelled rep, **b.** Cy3B free dye, c. Rep labelled at position 97C with Alexa Fluor 546 under 10 mM (red), 150 mM (orange) and 500 mM NaCl (blue) conditions. Solid black lines represent tri-exponential fits to the data and numbers (insets) are the amplitude weighted average lifetimes obtained under each condition. Intensity decays were obtained with excitation laser wavelength = 532 nm and emission detection peak wavelength = 572 nm under magic angle conditions. Number of technical replicates n = 3.

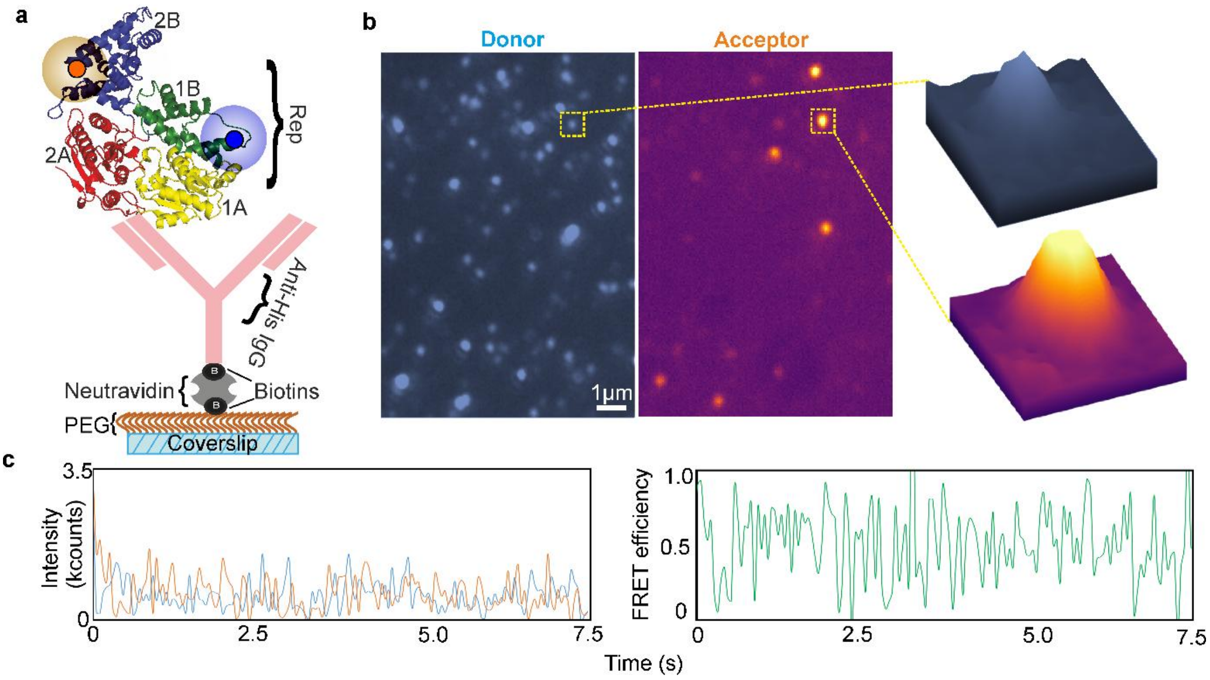

Supplementary Figure 4. Single-molecule fluorescence microscopy of surface-immobilized Rep shows impairment of Rep activity due to the closeness of the surface. **a.** Schematic of the immobilization of a single doubly labelled Rep conjugated via PEG-biotin/Neutravidin and Anti-His to the 6x His tag on Rep's N-terminus. It should be noted that an IgG is quite large with a typical Stokes radius of ~5 nm, so since Rep would then be typically at least ~10 nm from the surface there could still be some level of protein mobility within the TIRF evanescent field which could contribute to potential apparent noise. **b.** Representative widefield TIRF image of surface-immobilized Rep emitting in both donor (left) and acceptor (right) detector channels during donor-only excitation. Insets: zoom-ins of single immobilized foci. **c.** Representative donor (blur) and acceptor (orange) emission (left) with associated FRET efficiency (green, right) of a single Rep molecule displaying dynamic anti-correlated FRET behaviour.

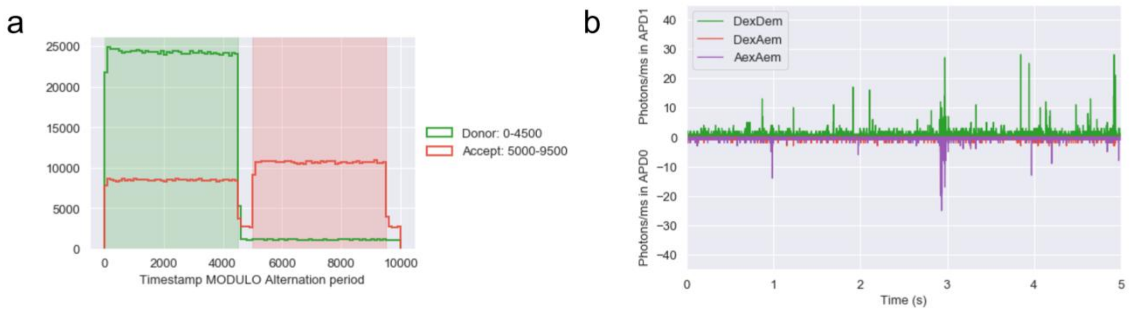

Supplementary Figure 5. Rapid µs alternating laser excitation (ALEX) can be used for excitation of the donor and acceptor separately. a. Histogram of photons by their arrival time within the ALEX cycle. The green laser is on during the green shaded area, and the red laser is on during the red shaded area. Green and red lines represent photons detected by the donor and acceptor channels. **b.** Representative example of raw µs data from confocal smFRET showing the donor and detector channels reconciled into coincident ms-timescale bursts.

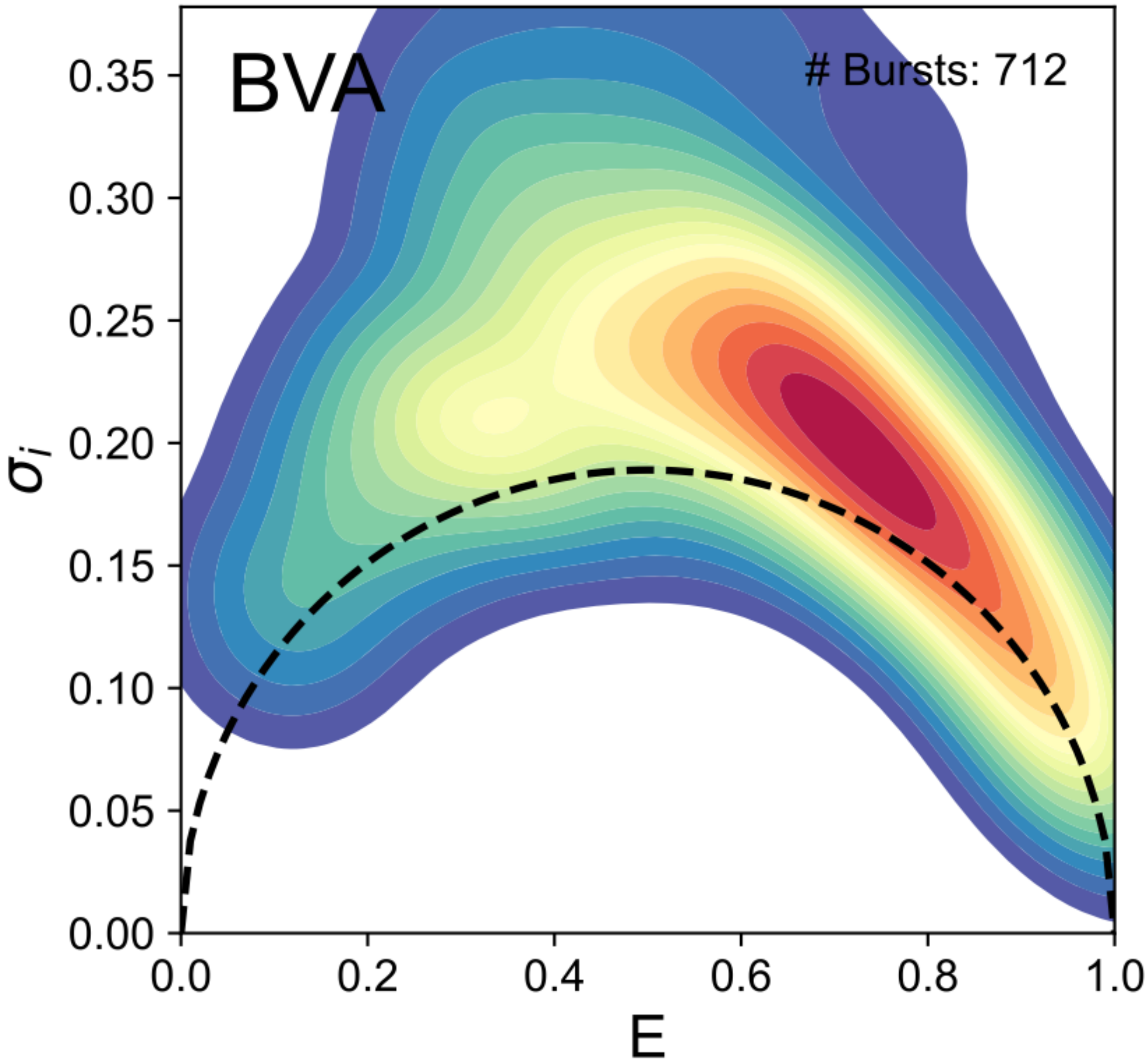

Supplementary Figure 6. Burst variance analysis. Heatmap showing burst variance analysis (BVA) on the low 10 mM NaCl data in the absence of DNA indicates that the bulk of the heatmap distribution of the SD of the inter-burst photon detection time (σ_i) was significantly high than theoretical expectations (black dashed line) for a system which does not undergo interconversion transitional kinetics(100), motivating subsequent quantitative H2MM analysis. Number of bursts = 712 obtained from low salt data.

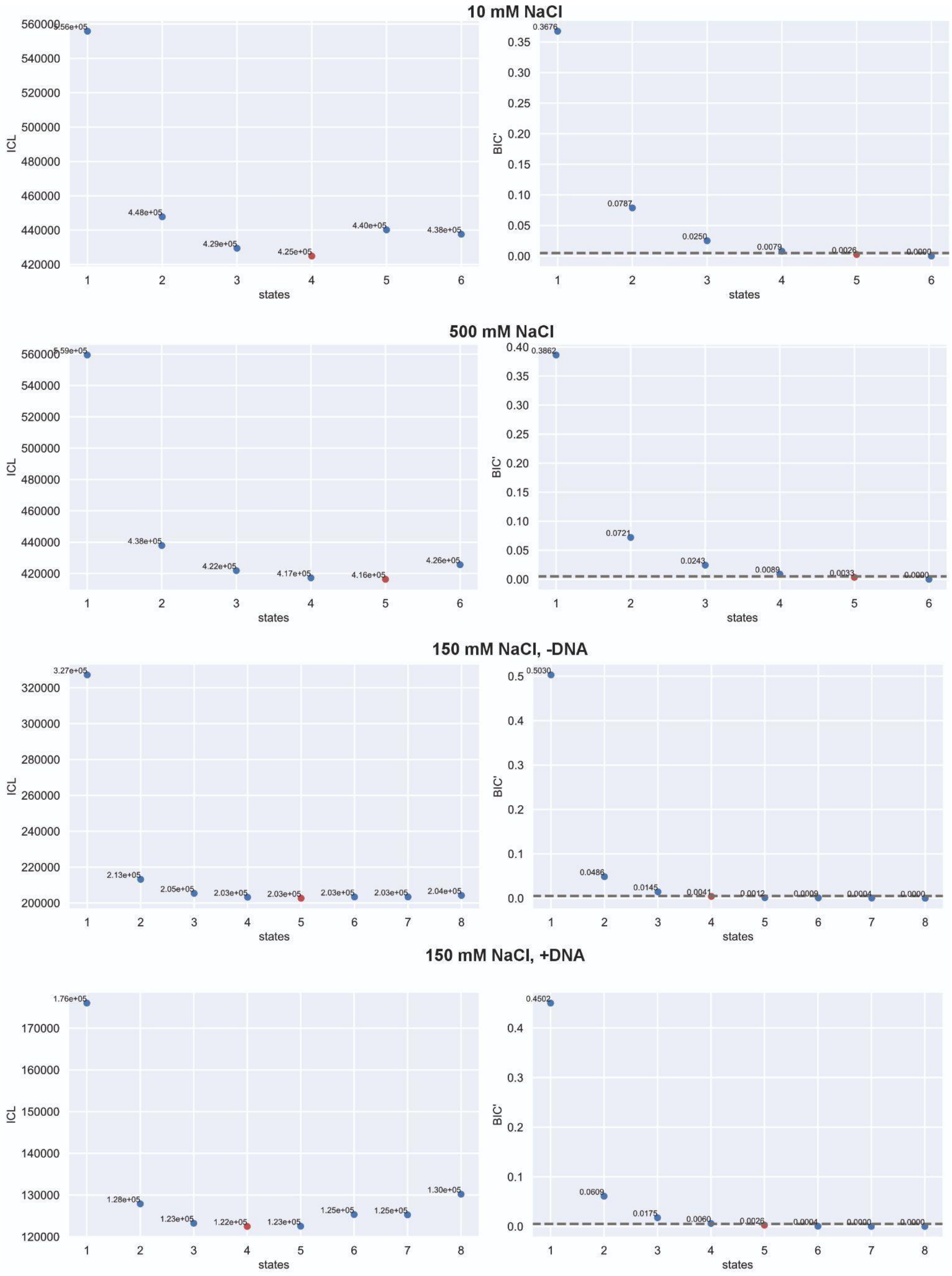

Supplementary Figure 7. Comparison of ICL and BIC' for H2MM. Plots indicating two different independent information criteria: ICL and modified BIC (BIC') as a function of the number of states in the H2MM models calculated (blue points). ICL results in a local minimum at the optimum model (red) for four or five states; further complexity from additional states is penalised and results in an increase. BIC' monotonically decreases and the optimal model is that which reaches a probability threshold of 0.005 (dashed line).

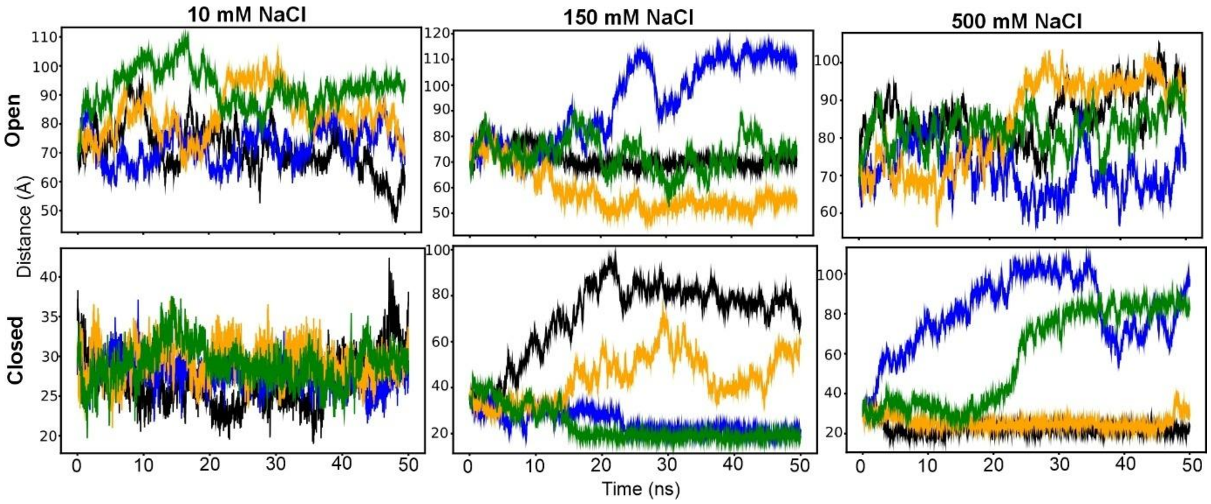

Supplementary Figure 8. MD simulation predictions of distance between FRET-labelled protein residues indicate open-closed state transitions in the absence of DNA. Time evolution of distance between residues 97 and 473 for each of the four replicates (different coloured lines) under each of the six conditions (low/intermediate/high salt and starting from open/closed conformations). Simulations initiated at the closed state and conducted at high salt were the only ones that do not show structural heterogeneity.

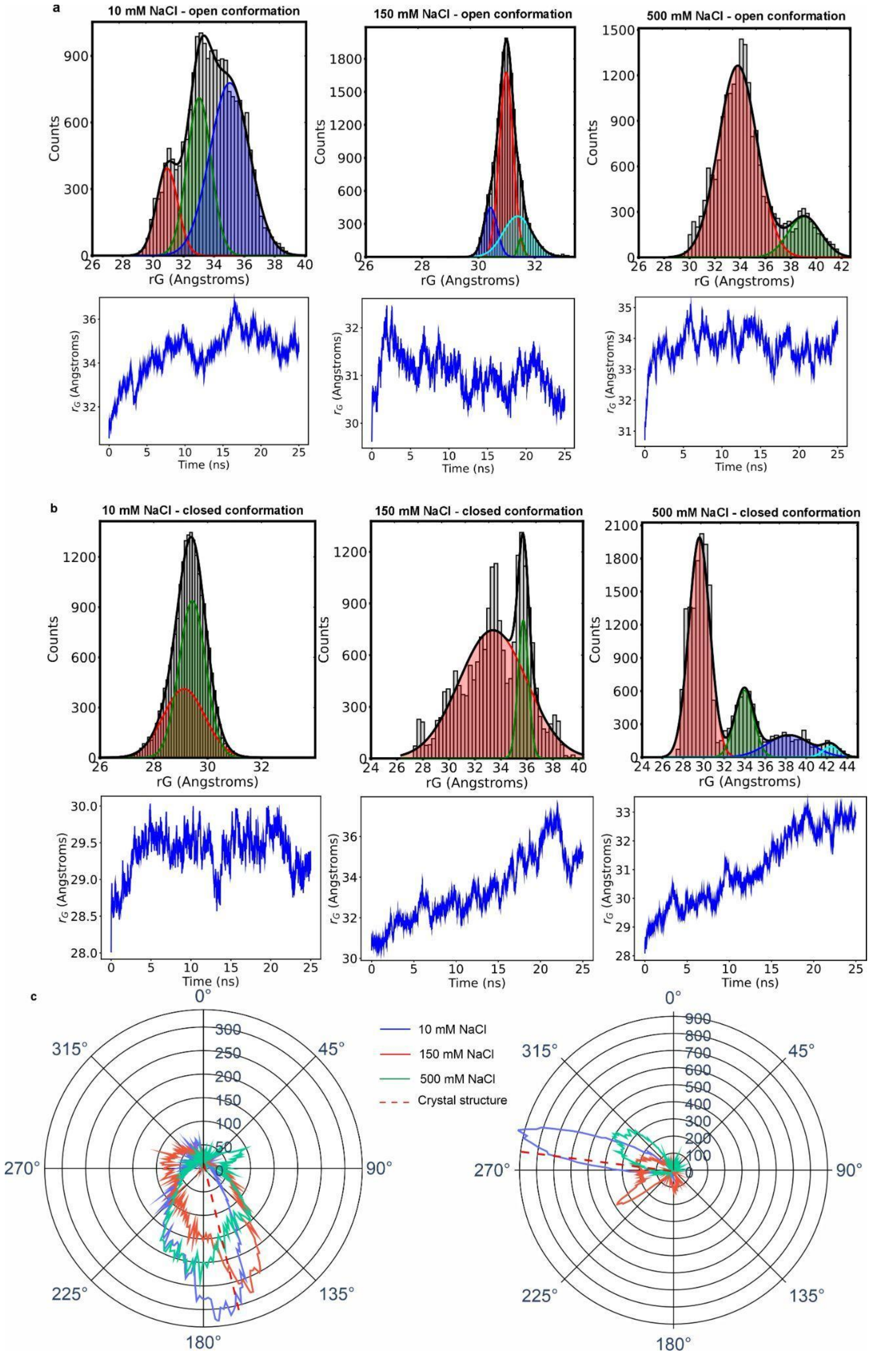
a
10 mM NaCl - open conformation
150 mM NaCl - open conformation
500 mM NaCl - open conformation
Counts
rG (Angstroms)
r_G (Angstroms)
Time (ns)
b
10 mM NaCl - closed conformation
150 mM NaCl - closed conformation
500 mM NaCl - closed conformation
c
10 mM NaCl
150 mM NaCl
500 mM NaCl
Crystal structure

Supplementary Figure 9. MDS predictions of free Rep radius of gyration and hinge rotation indicate structural heterogeneity. Molecular dynamics simulations starting either from **a.** open or **b.** closed Rep structures indicate a distribution of time-dependent radius gyration (rG) states whose distributions can be fitted with between 2-4 Gaussian functions (goodness of fit $R^2 \geq 0.93$ across all fits) depending on the level of NaCl concentration. Note, these Gaussian fits are performed free from any details of FRET levels. **c.** Population radar plots of the hinge rotation angle associated with WT-Rep initially in the open and closed conformations under 10 mM, 150 mM and 500 mM NaCl condition.

Supplementary Tables

Strain name	Gene and plasmid details	Reference
N6524	*pAM403 (lac$^+$ rep$^+$) / ΔlacIZYA::<>*	(11)
N6556	*pAM403 (lac$^+$ rep$^+$) / ΔlacIZYA::<> ΔuvrD::dhfr Δrep::cat*	(11)
HB222	*E. coli B F$^-$ ompT hsdS$_B$(r$_B^-$ m$_B^-$) gal dcm lon [malB$^+$]$_{K12}$(λ^S)*<br>*araB::T7RNAP-tetA Δrep::cat*	(33)

Supplementary Table 1. *Escherichia coli* K12 strains and plasmids used in this study.

**Optimised fitting parameters**	**NaCl 10 mM**	**NaCl 500 mM**	**NaCl 10 mM**	**NaCl 500 mM**	**NaCl 10 mM**	**NaCl 500 mM**
	**1-component model:**	**1-component model:**	**2-component model:**	**2-component model:**	**3-component model:**	**3-component model:**
a_1	5.81 ± 0.80	8.76 ± 0.14	1.17 ± 0.02	3.18 ± 0.09	0.79 ± 0.02	2.08 ± 0.14
τ_1 *(ns)*	1.34 ± 0.15	3.48 ± 0.03	2.99 ± 0.03	0.78 ± 0.03	3.46 ± 0.02	1.58 ± 0.33
a_2	--	--	9.15 ± 0.26	7.34 ± 0.04	5.15 ± 0.16	6.78 ± 0.28
τ_2 *(ns)*	--	--	0.56 ± 0.01	3.71 ± 0.01	0.87 ± 0.02	3.81 ± 0.04
a_3	--	--	--	--	8.26 ± 0.49	2.52 ± 0.12
τ_2 *(ns)*	--	--	--	--	0.26 ± 0.02	0.35 ± 0.08
τ_{av} *(ns)*	1.34 ± 0.15	3.48 ± 0.03	1.55 ± 0.01	3.47 ± 0.01	1.49 ± 0.01	3.47 ± 0.01
**Reduced χ^2**	36.4	4.88	2.29	1.27	1.42	1.17

Supplementary Table 2 Fitting parameters corresponding to mono, bi- and tri-exponential models used to evaluate the fluorescence lifetime of doubly labelled Rep in low and high salt buffers. Errors are reported as errors of the fit. Convergence of the reduced χ^2 parameter was used to evaluate that the fluorescence decays exhibited tri-, rather than mono- or bi-exponential behaviour.

Optimised fitting parameters	10 mM NaCl	500 mM NaCl	150 mM NaCl	150 mM NaCl + DNA
**4-component model:**				
**Weight 1**	11.0	24.5	18.0	13.8
**Weight 2**	23.3	24.9	15.5	22.2
**Weight 3**	47.4	33.2	30.3	31.4
**Weight 4**	18.3	17.4	36.1	32.6
$<E_1> \pm \sigma$	0.21 ± 0.18	0.21 ± 0.13	0.11 ± 0.12	0.11 ± 0.11
$<E_2> \pm \sigma$	0.54 ± 0.12	0.54 ± 0.12	0.44 ± 0.14	0.44 ± 0.14
$<E_3> \pm \sigma$	0.82 ± 0.09	0.82 ± 0.09	0.8 ± 0.11	0.8 ± 0.10
$<E_4> \pm \sigma$	0.98 ± 0.02	0.98 ± 0.02	0.97 ± 0.03	0.97 ± 0.03
Goodness of fit R^2	0.91	0.88	0.77	0.80
Reduced χ2	1.89		3.21	
BIC	-234.59		-354.34	
**3-component model:**				
$<E_1> \pm \sigma$	0.23 ± 0.19	0.23 ± 0.14	0.23 ± 0.19	0.23 ± 0.18
$<E_2> \pm \sigma$	0.60 ± 0.15	0.60 ± 0.13	0.76 ± 0.12	0.76 ± 0.14
$<E_3> \pm \sigma$	0.89 ± 0.09	0.89 ± 0.08	0.97 ± 0.03	0.97 ± 0.03
Goodness of fit R^2	0.66	0.52	0.76	0.78
Reduced χ2	6.51		4.46	
BIC	-82.98		-292.96	
**2-component model:**				
$<E_1> \pm \sigma$	0.42 ± 0.23	0.42 ± 0.23	0.47 ± 0.30	0.47 ± 0.28
$<E_2> \pm \sigma$	0.87 ± 0.11	0.87 ± 0.10	0.95 ± 0.05	0.95 ± 0.05
Goodness of fit R^2	0.73	0.59	0.63	0.67
Reduced χ2	7.40		7.75	
BIC	-51.77		-157.61	

Supplementary Table 3 Optimized parameters for Gaussian fits for smFRET using confocal microscopy on labelled Rep in low (10 mM) and high (500 mM) NaCl as well as an intermediate NaCl concentration (150 mM) ± DNA. Taken using number of bursts n = 1,021 and 1,013 from high and low salt respectively, from 7 different samples, for n = 1,141 from 9 different samples for intermediate salt, and for n = 1250 from 10 different samples for intermediate salt + DNA. <Ei> value is the mean FRET efficiency values for the ith Gaussian curve in the fit. Corresponding mean FRET efficiencies with R^2, reduced Chi squared and BIC metrics shown for 4-, 3- and 2-component models, all of which show that the 2- and 3-component models clearly exhibit worse fits than the 4-component model. The addition of a 5th component resulted in convergence to one of the existing 4 components and demonstrated a less negative BIC metric than the 4-componet model, thus supporting a 4-component best fit model.

	10 mM NaCl		500 mM NaCl		150 mM NaCl		150 mM NaCl + DNA	
	FRET Efficiency	Relative Occupancy	FRET Efficiency	Relative Occupancy	FRET Efficiency	Relative Occupancy	FRET Efficiency	Relative Occupancy
**S1**	0.2050 ± 0.0031	35.72%	0.2067 ± 0.0021	32.28%	0.2388 ± 0.0038	17.41%	0.2696 ± 0.0046	25.84%
**S2**	0.5705 ± 0.0036	31.95%	0.4911 ± 0.0034	23.28%	0.5114 ± 0.0059	26.29%	0.5648 ± 0.0065	24.27%
**S3**	0.7292 ± 0.0021	22.72%	0.7212 ± 0.0025	27.23%	0.8093 ± 0.0038	29.66%	0.8108 ± 0.0048	25.69%
**S4**	0.9454 ± 0.0013	9.61%	0.9441 ± 0.0013	17.21%	0.9599 ± 0.0015	26.65%	0.9585 ± 0.0017	24.20%

			Transition Rate (s^{-1}) [lower bound, upper bound]			
			End State			
			**S1**	**S2**	**S3**	**S4**
**10 mM NaCl**	**Start State**	**S1**		29005.55 [28355.78, 29976.38]	958.31 [918.36, 999.33]	28.00 [13.39, 44.14]
		**S2**	25069.40 [24479.10, 26015.52]		5.19 [0.00, 35.25]	0.00 [0.00, 8.02]
		**S3**	390.08 [373.38, 407.38]	28.71 [11.38, 44.77]		306.27 [292.01, 320.98]
		**S4**	0.00 [0.00, 4.54]	0.00 [0.00, 2.46]	463.88 [441.08, 487.27]	
**500 mM NaCl**	**Start State**	**S1**		951.63 [912.67, 991.60]	0.00 [0.00, 6.67]	74.28 [64.23, 84.93]
		**S2**	1003.92 [959.82, 1049.31]		367.50 [344.56, 391.18]	0.00 [0.00, 4.83]
		**S3**	14.70 [6.48, 23.98]	344.98 [327.13, 363.50]		312.05 [294.52, 330.17]
		**S4**	33.95 [24.30, 44.54]	0.00 [0.00, 9.07]	437.46 [413.86, 461.81]	
**150 mM NaCl**	**Start State**	**S1**		778.04 [690.92, 871.28]	17.03 [0.00, 63.33]	0.00 [0.00, 16.42]
		**S2**	943.71 [853.24, 1039.67]		1296.72 [1186.22, 1412.58]	0.00 [0.00, 31.13]
		**S3**	0.00 [0.00, 21.69]	1011.81 [929.22, 1098.82]		611.48 [545.13, 681.60]
		**S4**	0.00 [0.00, 5.62]	0.00 [0.00, 9.79]	544.14 [485.49, 606.10]	
**150 mM NaCl + DNA**	**Start State**	**S1**		1262.28 [1154.72, 1375.06]	0.00 [0.00, 30.25]	0.00 [0.00, 8.31]
		**S2**	1572.31 [1440.58, 1711.40]		945.39 [843.48, 1053.97]	11.43 [0.00, 55.65]
		**S3**	0.00 [0.00, 20.38]	883.52 [793.22, 979.14]		1096.49 [989.05, 1209.74]
		**S4**	13.12 [0.00, 35.50]	0.00 [0.00, 30.24]	948.53 [861.62, 1039.51]	

Supplementary Table 4 Optimised parameters from H2MM analysis. Upper panel shows the optimised outputs for the relative occupancy of the four states. Lower panel indicates the optimised interconversion rate constants between all four states. Errors and low/upper limits quoted are based on log-likelihood uncertainty estimation (see Methods).

State interconversion transitions	Rep				UvrD
	k (s^{-1}) *in 10mM NaCl*	*k* (s^{-1}) *in 150mM NaCl, no DNA*	*k* (s^{-1}) *in 150mM NaCl, +DNA*	*k* (s^{-1}) *in 500 mM NaCl*	*k* (s^{-1}) *in 60 mM NaCl*
S1 to S2	>>1,000	1,262	1,572	952	0.85
S2 to S1	>>1,000↔	945↔	1,262↔	1,004↔	0.26↓
S2 to S3	5	1,297	945	368	0.30
S3 to S2	29↑	1,021↔	884↔	345↔	0.25↓
S3 to S4	306	611	1096	312	
S4 to S3	464↔	544↔	948↔	437↔	0.20↓
S1 to S3	958	<1	<1	< 1	0.16
S3 to S1	390↓	<1	<1	15↑	0.18↔
S2 to S4	<1	<1	11↑	<1	0.54
S4 to S2	<1↔	<1	<1	<1↔	0.33↓
S4 to S1	<1	<1	13↑	34	--
S1 to S4	28↑	<1	<1	74↑	--

Supplementary Table 5 Comparison of transitional kinetics rate constants for states previously identified for UvrD(23) and those measured in our study for Rep at the high and low levels. Yellow highlight indicates > 2x difference between rates at low (10 mM) and intermediate (150 mM) and/or high (500 mM) NaCl concentration for Rep in absence of DNA; ↓ indicates rate between states SY to SX relative to SX to SY decreases; ↔ indicates rates between SY to SX rate remains unchanged within a factor of 2 relative to rates between states SX to SY; ↑ indicates rate between states SY to SX increases relative to rate between states SX to SY. Rate constants at 150 mM NaCl in presence of DNA are also included for reference.

Parameter	Alexa Fluor 546 (donor)	Alexa Fluor 647 (acceptor)
Linker length (Å)	14	14
Linker width (Å)	4.5	4.5
Radius 1 (Å)	6.8	11
Radius 2 (Å)	3.9	3
Radius 3 (Å)	1.8	1.5
R_0 (Å)	68.2	

Supplementary Table 6. Fluorophores with their respective linkers that were implemented onto modelling structures of Rep.

Supplementary movie screenshots and legends

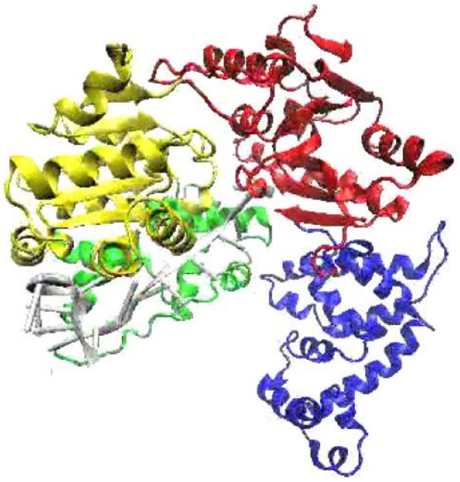

Supplementary Movie 1 MD simulation (starting from open conformation), Rep with DNA bound, low salt (10 mM NaCl), implicit solvent.

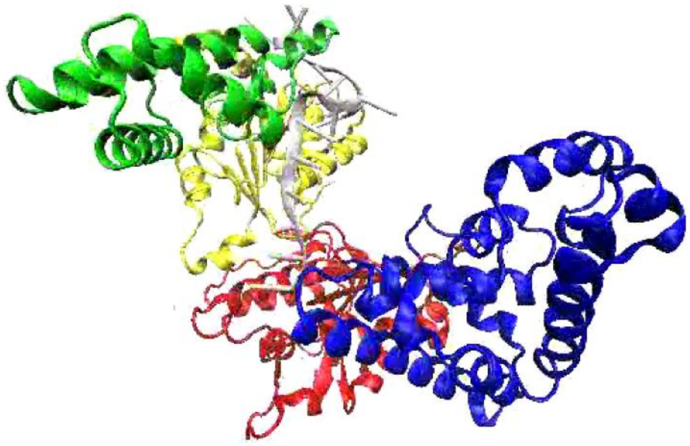

Supplementary Movie 2 MD simulation (starting from open conformation), Rep with DNA bound, high salt (500 mM NaCl), implicit solvent.

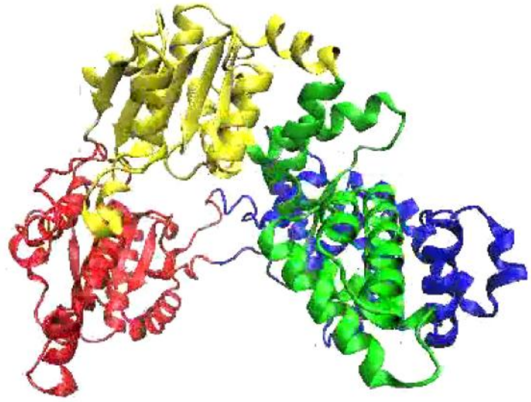

Supplementary Movie 3 MD simulation (starting from open conformation), Rep with no DNA, low salt (10 mM NaCl), implicit solvent.

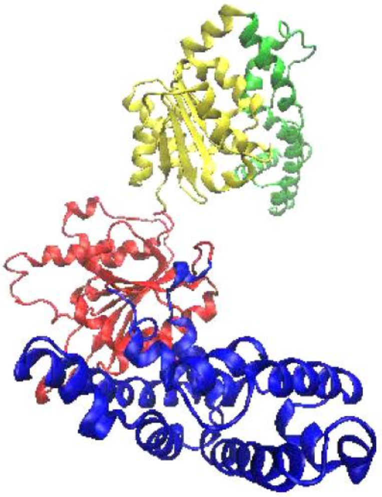

Supplementary Movie 4 MD simulation (starting from open conformation), Rep with no DNA, high salt (500 mM NaCl), implicit solvent.

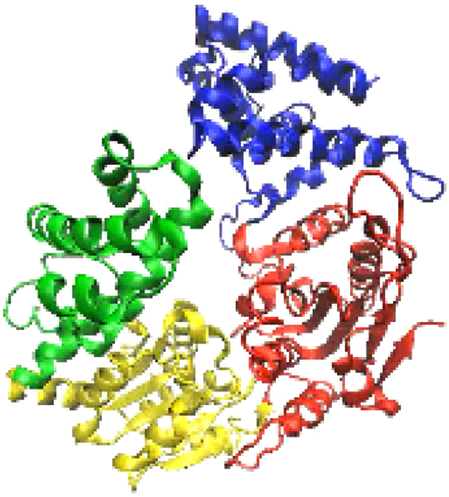

Supplementary Movie 5 MD simulation (starting from open conformation), Rep with no DNA, intermediate salt (150 mM NaCl), implicit solvent.

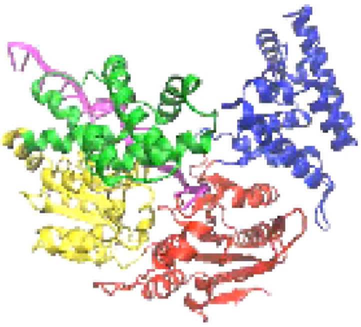

Supplementary Movie 6 MD simulation (starting from open conformation), Rep with DNA (shown in magenta), intermediate salt (150 mM NaCl), implicit solvent.

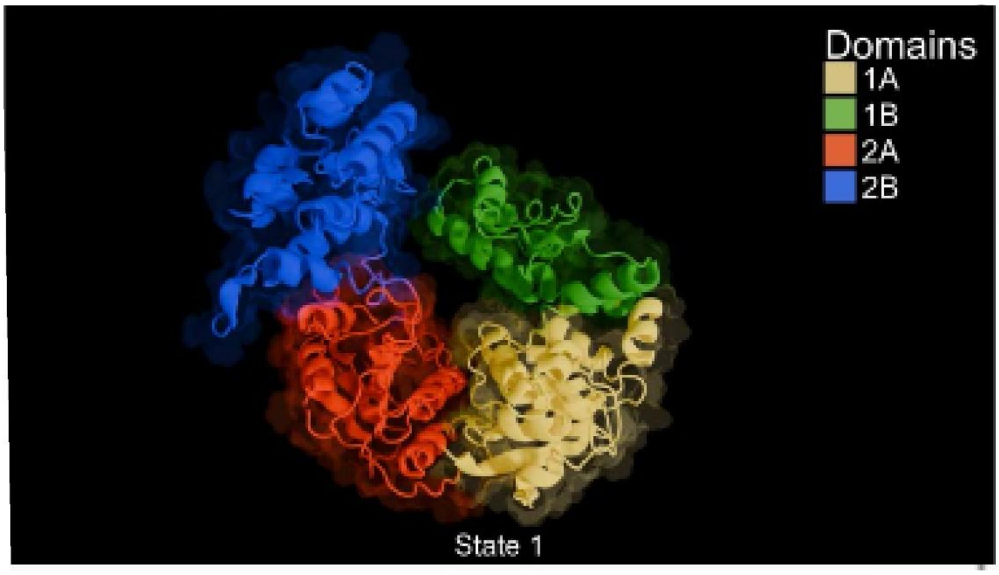

Supplementary Movie 7 Animation depicting structural transitions between states S1, S2, S3 and S4.
